# Downregulation of MicroRNA-206 Alleviates the Sublethal Oxidative Stress-Induced Premature Senescence and Dysfunction in Mesenchymal Stem Cells via Targeting Alpl

**DOI:** 10.1155/2020/7242836

**Published:** 2020-02-13

**Authors:** Xuan Liu, Ziying Yang, Qingyou Meng, Yueqiu Chen, Lianbo Shao, Jingjing Li, Yihuan Chen, Zhenya Shen

**Affiliations:** Department of Cardiovascular Surgery of the First Affiliated Hospital & Institute for Cardiovascular Science, Soochow University, Suzhou, China

## Abstract

Bone marrow-derived mesenchymal stem cells (MSCs) have shown great promise in tissue engineering and regenerative medicine; however, the regenerative capacity of senescent MSCs is greatly reduced, thus exhibiting limited therapy potential. Previous studies uncovered that microRNA-206 (miR-206) could largely regulate cell functions, including cell proliferation, survival, and apoptosis, but whether miR-206 is involved in the senescent process of MSCs remains unknown. In this study, we mainly elucidated the effects of miR-206 on MSC senescence and the underlying mechanism. We discovered that miR-206 was upregulated in the senescent MSCs induced by H_2_O_2_, and abrogation of miR-206 could alleviate this tendency. Besides, we determined that by targeting Alpl, miR-206 could ameliorate the impaired migration and paracrine function in MSCs reduced by H_2_O_2_. In vivo study, we revealed that inhibition of miR-206 in senescent MSCs could effectively protect their potential for myocardial infarction treatment in a rat MI model. In summary, we examined that inhibition of miR-206 in MSCs can alleviate H_2_O_2_-induced senescence and dysfunction, thus protecting its therapeutic potential.

## 1. Introduction

Bone marrow-derived mesenchymal stem cells (MSCs), which can be easily isolated [1], have been widely studied in tissue engineering and regenerative medicine due to their capacity of immunomodulatory effects, self-renewal, and multilineage differentiation [2]. In addition, they are also an optimal candidate in cell therapy strategies due to their feasible and safer properties in regard to the risk of forming tumors and becoming cancerous [3]. However, MSCs have to undergo senescence during the replicative process or when exposed to oxidative stress, which in turn greatly impairs their regenerative capacity and limits their transplantation efficiency [4, 5].

Thus, to research the molecular procedures controlling MSCs, senescence is not only pivotal to verify the mechanism of age-associated MSC dysfunction but critical for improving or even reversing the therapeutic effect of senescent MSCs, so as to provide an optimum therapeutic strategy for age-associated diseases.

MicroRNAs are a class of noncoding RNAs that can regulate their target gene expression through interfering with posttranscriptional pathways by degrading mRNA or inhibiting protein translation [6]. Among those well-studied microRNAs, miR-206 belongs to the miR-1/miR-206 family and has been detected in many tissues/cell types, such as the brain [7], skeletal muscles [8, 9], hearts [10], cancer cell lines [11, 12], and brown adipocytes [13]. It was reported that miR-206 could largely regulate cell functions. For example, Wang et al. have found that overexpression of miR-206 significantly inhibited the proliferation and promoted apoptosis at the early stages in glioma cells and neuroblastoma cells while downregulation of it notably enhanced the proliferation capacity and suppressed cell apoptosis [14]. Hesari et al. have confirmed that upregulation of miR-206 attenuated cell survival and promoted apoptosis in breast cancer cells [15]. In addition, Wang and Tian have reported that miR-206 inhibited cell proliferation, migration, and invasion in human cervical cancer cells [11]. These studies indicate that miR-206 acts as an important modulator in the physiological process of cells. However, whether miR-206 is involved in the senescent process of MSCs and by which molecular mechanisms remain unknown.

Considering limited studies reported about the association between miR-206 and MSC senescence, the present study was conducted to elucidate effects of miR-206 on the senescent process of MSCs as well as the underlying mechanisms.

## 2. Methods and Materials

### 2.1. Animals

SD female rats were purchased from the Experiment Animal Center of Soochow University (Suzhou, China). All the procedures were performed according to the guidelines of the Institutional Animal Care and Use Committee of Soochow University.

Animals were housed on a 12-hour light/dark cycle and provided with standard laboratory food and water.

### 2.2. Culture and Characterization of MSCs

Bone marrow-derived mesenchymal stem cells (MSCs) were collected as described previously [16]. In brief, MSCs were flushed out from the femur of the SD rat using DMEM/F12 medium (Gibco, China), then centrifuged, suspended, and planted onto a 6 cm diameter plate. Cells were cultured with DMEM/F12 medium supplemented with 10% fetal bovine serum and 1% penicillin/streptomycin in a humidified cell culture incubator at 37°C with 5% CO_2_. The cells were dissociated by 0.25% trypsin-EDTA (Invitrogen) and passaged at 1 : 3 dilution when reaching 90% confluence. The following antibodies, PE-conjugated anti-CD90 and anti-CD45, Alexa488-conjugated anti-CD29, and FITC-conjugated anti-CD11b, anti-CD34, and anti-CD86 of MSCs, were used to identify the characteristic properties of MSCs using flow cytometry (Millipore Guava easyCyte). All subsequent assays were performed with cells at passages 3 to 5.

### 2.3. H_2_O_2_ Treatment and Cell Transfection

The miR-206 mimic, inhibitor, and negative control were purchased from GenePharma Co., Ltd. (Shanghai, China) and dissolved with diethylpyrocarbonate- (DEPC-) treated water to 200 nM. Cell transfection was performed with Lipofectamine 2000 (Invitrogen, Carlsbad, CA, USA) according to the manufacturer's description. Alpl inhibitor (levamisole, Sigma) with concentration of 100 *μ*mol/L was used to inhibit Alpl expression. Briefly, the cells were firstly treated with H_2_O_2_ at a concentration of 200 *μ*M for 2 hours [17]. After washing with serum-free medium for three times, cells were then transfected with miR-206 inhibitor, followed by levamisole treatment or not for 48 hours. The experimental groups were thus divided as follows: NC group (normal MSCs), H_2_O_2_ group (MSCs with H_2_O_2_ treatment), miR-206 inhibitor group (MSCs treated with H_2_O_2_ and miR-206 inhibitor), and miR-206 inhibitor+levamisole group (MSCs treated with H_2_O_2_ and miR-206 inhibitor+levamisole). After 48 hours, the cells were used for further assays.

### 2.4. SA-*β*-gal Staining

MSC senescence was determined by using the *β*-galactosidase (SA-*β*-gal) staining kit (Beyotime, China) according to the manufacturer's instructions. The cells were incubated overnight at 37°C without CO_2_, and cell nuclei were counterstained with DAPI later. The number of SA-*β*-gal-positive cells was determined in 10 randomly chosen fields, and a total of at least 200 cells from each sample were counted.

### 2.5. Apoptosis Assay

The cell apoptosis was detected using an Annexin V-PE/7-AAD detection apoptosis kit (FCMACS, China) according to the manufacturer's instructions. Briefly, a total of 1 × 10^6^ cells were collected and resuspended in the mixture containing Annexin V-PE and 7-AAD for incubation in the dark. After 15 min, the apoptosis of MSCs was analyzed using a flow cytometry.

### 2.6. Relative Telomere Length

DNA samples were extracted from MSCs using a TIANamp Genomic DNA kit (Qiagen Sciences Inc., Germantown, MD). The determination of relative telomere length was performed as previously described [18]. Quantitative PCR was performed using the StepOnePlus Detection system (Applied Biosystems, USA) according to the manufacturer's instructions. The single copy gene AT1 was used as an internal reference; the ratio of telomere repeat copy number and single gene copy number was regarded as the relative telomere length.

### 2.7. ELISA Assay

The level of inflammatory factors including TNF-*α* and IL-1*β* in the medium of cultured cells was determined using ELISA kits (Abcam, UK) according to the manufacturer's instructions.

### 2.8. EdU Staining Assay

The EdU staining assay was performed using the BeyoClick™ EdU Cell Proliferation Kit with Alexa Fluor 555 (Beyotime, China) according to the manufacturer's instructions. Briefly, the cells were cocultured with 10 *μ*M EdU for 2 hours and washed later with PBS three times, followed by 4% paraformaldehyde fixation. Then, cells were incubated with permeable solution and washed with PBS three times. Next, cells were incubated with the Click Additive Solution for 30 min and washed with PBS followed by incubation with Hoechst 33342 staining solution at room temperature for 15 min. Images of the staining were captured with a fluorescent microscope (Olympus, Tokyo, Japan).

### 2.9. Transwell Migration Assay

The transwell migration assay was performed using transwell filters with 8 *μ*m pores (Fisher Scientific, Pittsburgh, PA, USA). Briefly, cells (5 × 10^4^ cells/200 *μ*l) suspended in serum-free DMEM were planted into the upper compartment of a transwell chamber while lower chambers were filled with 500 *μ*l of DMEM containing 10% FBS. After 4 hours, cells were fixed in 4% methanal for 20 min, stained with DAPI (Invitrogen) for 15 min, and counted under a fluorescent microscope (Olympus, Tokyo, Japan).

### 2.10. Tube Formation Assay

Culture supernatants in different groups were collected, and HUVECs were cultured with these cell supernatants for 24 h. Then, the pretreated HUVECs were seeded on the Matrigel at 2 × 10^4^ cells/well and incubated at 37°C for 6 hours. Finally, images were photographed under an inverted microscope and evaluated by ImageJ software.

### 2.11. Dual-Luciferase Reporter Assay

TargetScan (http://targetscan.org) was used to predict whether Alpl was a target gene of miR-206. The wild-type- (wt-) 3′-untranslated region (UTR) of Alpl and mutant- (mut-) 3′-UTR of Alpl in which miR-206 might bind were cloned into pisCHECK2 vector (Thermo Fisher Scientific Inc., USA). NC or miR-206 mimic was transfected into HEK293T cells (ATCC, Manassas, VA, USA) with either Alpl-wt or Alpl-mut luciferase reporter plasmids. After cultivation at 37°C for 24 hours, the Dual-Luciferase Reporter Assay kit (Promega, Madison, USA) was applied to detect luciferase activity according to the manufacturer's instructions.

### 2.12. Acute Myocardial Infarction Model and Cardiac Function Assessment

Acute myocardial infarction (AMI) was induced according to the method described previously [19]. Briefly, young female SD rats (~250 g) were anesthetized with 10% chloral hydrate (*w*/*v*; Acros, Japan) by intraperitoneal injection. The left anterior descending artery was ligated with a 6–0 prolene suture between the pulmonary artery outflow tract and the left atrium. Then, a total of 1 × 10^6^ MSCs in 20 *μ*l of phosphate-buffered saline (PBS) were transplanted by myocardial injection near the ligation site (3–4 sites/heart) in the free wall of the left ventricle. Rats under operation were randomly divided into four groups: PBS group (PBS, *n* = 5), NC group (normal MSCs, *n* = 5), H_2_O_2_ group (MSCs treated with H_2_O_2_, *n* = 5), and miR-206 inhibitor group (MSCs treated with H_2_O_2_ followed by miR-206 inhibitor treatment, *n* = 5).

Echocardiography was conducted 28 days after MSCs transplantation to detect cardiac function using the Vevo 2100 system (VisualSonics Inc., Toronto, ON, Canada). The left ventricular ejection fraction (EF) and fraction shorting (FS) were calculated according parameters recorded from two-dimensional images using the M-mode interrogation in the short-axis view. Then, the rats were euthanized to collect the heart tissue samples for histological examination.

### 2.13. Histological Examination

The isolated fresh heart tissues were fixed in 4% paraformaldehyde and embedded in paraffin, then specimens were cut into 5 *μ*m thick slices for Masson trichrome (MT) staining as described previously [20]. The section stained blue was considered to be myocardial fibrosis, and the severity of myocardial fibrosis was analyzed by ImageJ software (National Institutes of Health, USA). For immunofluorescence staining of CD31, the sections were first incubated with primary antibody CD31 overnight at 4°C. Then, secondary antibody donkey anti-mouse FITC 488 (1 : 500; Life Technologies) were incubated at room temperature for 1 hour. After washing, the nuclei were counterstained with DAPI later. Images were observed with an inverted fluorescence microscope (Olympus).

### 2.14. Quantitative Real-Time PCR Assay

Total RNA in each sample was isolated using a TRIzol reagent (Invitrogen, USA). The reverse transcription of miR-206 was performed by the PrimeScript RT Reagent Kit (Takara, Japan). Q-PCR was carried out to detect gene expression level in the ABI StepOnePlus detection system (Applied Biosystems, USA) according to the manufacturer's introductions. The relative gene expression quantifications were evaluated according to the 2^-*ΔΔ*CT^ method, with U6 and GAPDH acting as the internal control. The mean values were presented as the gene expression levels. Primers used in the study are presented in Table 1.

### 2.15. Western Blot Analysis

For western blot analyses, cells were lysed in ice-cold cell lysis buffer (Beyotime, China) containing protease inhibitors, with the concentration of extracted protein detected by a BCA Protein Assay Kit (Beyotime, China). Then, equal amounts of protein (40 *μ*g) from each group were loaded and separated by 10% SDS-PAGE and transferred to polyvinylidene difluoride (PVDF) membranes. Thereafter, the membranes were blocked using Tween 20/PBS including 5% skim milk and incubated with a primary antibody (Abcam, Cambridge, MA, USA) at 4°C overnight, followed by incubating in secondary antibodies (Abcam, Cambridge, MA, USA) at room temperature for 1 hour. After washing, the protein-labeled bands were determined using the enhanced chemiluminescent kit and analyzed using the Scion ImageJ software (National Institutes of Health, USA).

### 2.16. Statistical Analysis

All data were analyzed using GraphPad Prism 7.0 software (GraphPad Software, San Diego, CA, USA). Statistical differences between two groups were determined by Student's unpaired *t*-test, whereas statistical differences among three or more groups were determined by one-way ANOVA test. *P* < 0.05 was considered as statistical significance.

## 3. Results

### 3.1. Characterization of Rat MSCs

**Figure 1:**
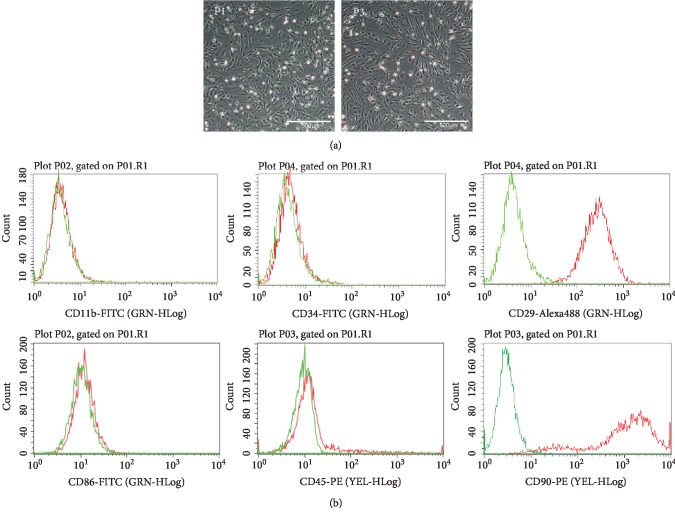
Characterization of rat MSCs. (a) Morphology of MSCs at passage 1 and passage 3 observed under an inverted microscope. (b) Phenotypic analysis of cell surface marker antigens of MSCs by flow cytometry.

After flushing out from the femur of SD rats and being cultured in a humidified incubator, the cells were adherent to the plastic dishes and showed a typical spindle shape (Figure 1(a)). Besides, flow cytometry showed that the cultured cells were positive for CD90 and CD29 and negative for CD34, CD11b/c, CD86, and CD45 (Figure 1(b)), which was consistent with the surface markers of MSCs. These results indicated that the isolated and cultured cells were MSCs and could be used for further experiments.

### 3.2. Treatment with H_2_O_2_ Successfully Induced MSC Senescence

**Figure 2:**
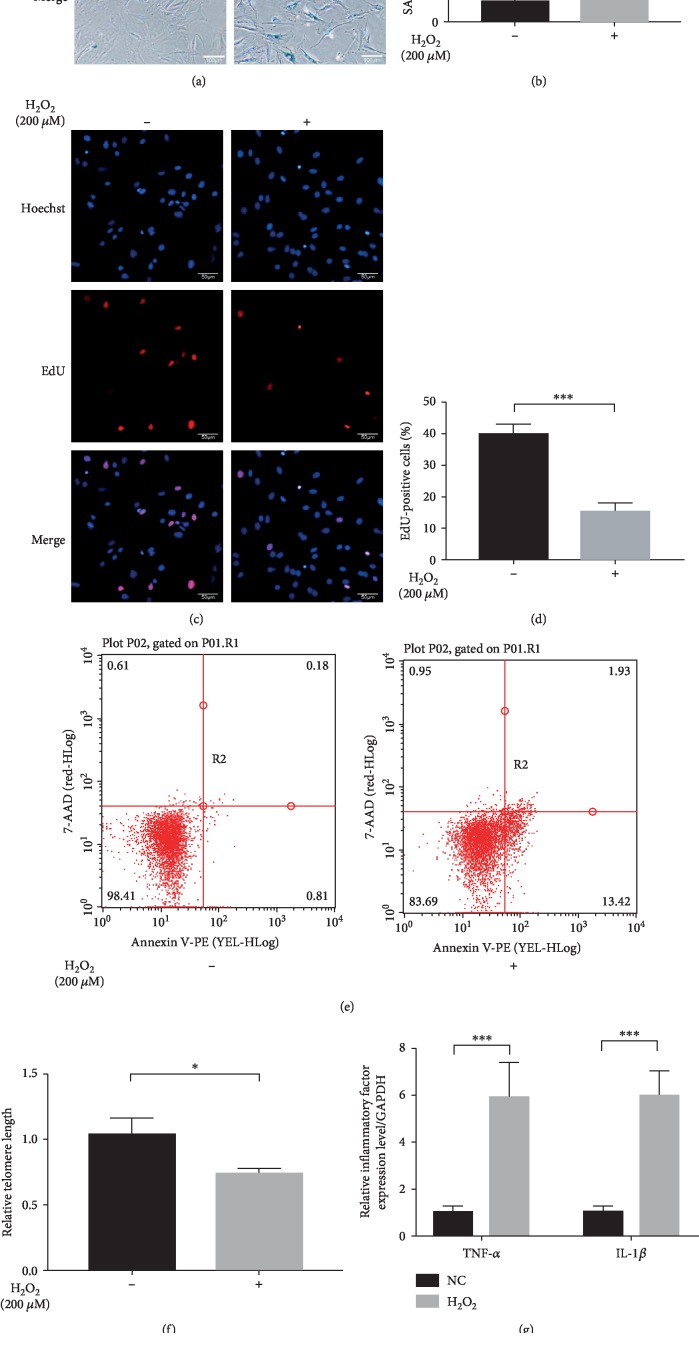
H_2_O_2_ treatment induced senescence of MSCs. MSCs under H_2_O_2_ treatment showed increased percentage of SA-*β*-gal staining cells (*n* = 4) (a, b), reduced percentage of EdU-positive staining cells (*n* = 4) (c, d), expanded percentage of early apoptotic cells (*n* = 3) (e), shorter relative telomere length (f), and upregulated inflammatory factor (TNF-*α* and IL-1*β*) expressions (*n* = 3) (g, h) in contrast to normal MSCs. (i) The expression of miR-206 was highly expressed in MSCs under H_2_O_2_ treatment than in normal MSCs (*n* = 3). ^∗^*P* < 0.05, ^∗∗^*P* < 0.01 and ^∗∗∗^*P* < 0.001.

To induce MSC senescence, cells were treated with H_2_O_2_ as previously described [17]. We discovered that the percentage of blue staining cells increased significantly (Figures 2(a) and 2(b)) whereas the percentage of EdU staining cells decreased notably in the H_2_O_2_ group than in the NC group (Figures 2(c) and 2(d)). Besides, MSCs under H_2_O_2_ treatment depicted higher percentage of early apoptotic cells (Figure 2(e)) and shorter relative telomere length (Figure 2(f)). What is more, inflammatory factors such as TNF-*α* and IL-1*β* were also dramatically upregulated in H_2_O_2_-treated MSCs than in normal MSCs (Figures 2(g) and 2(h)), which was consistent with a previous study [21]. Collectively, these results suggested that the senescent type of MSCs was successfully constructed. Then, we examined the expression level of miR-206 by Q-PCR analysis; the results presented in Figure 2(i) showed that the expression of miR-206 was remarkably upregulated in the senescent MSCs compared to normal MSCs.

### 3.3. Alpl Was a Target Gene of miR-206

**Figure 3:**
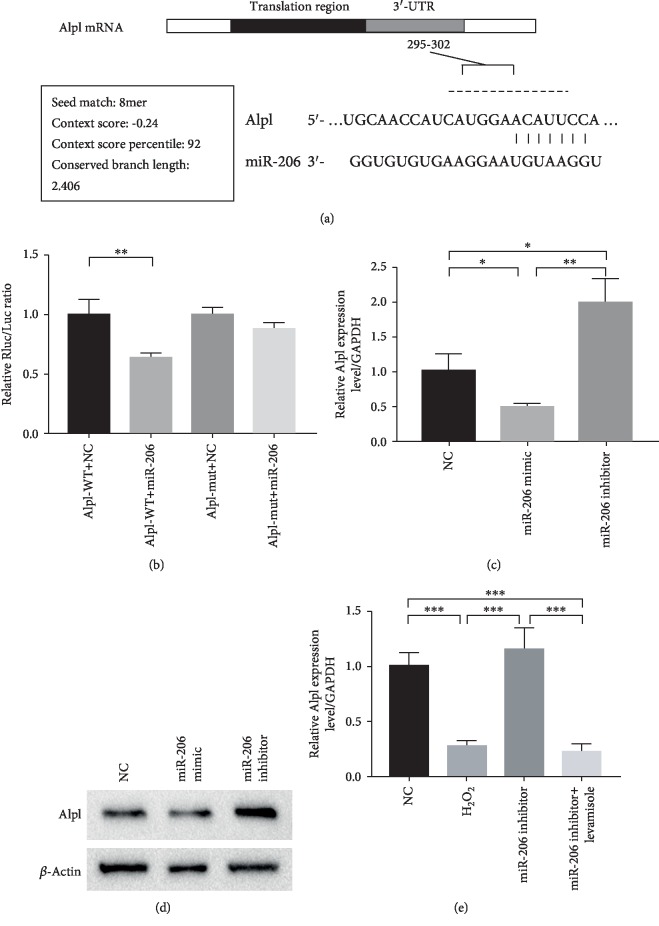
Alpl was a target gene of miR-206. (a) The binding segments of miR-206 on the 3′-UTR of Alpl (295-302). (b) Relative Rluc/Luc ratio (*n* = 3) (c). The mRNA (c) and protein (d) expression level of Alpl in MSCs after transfection of miR-206 mimic and inhibitor, respectively (*n* = 3). (e) Expression levels of Alpl in untreated MSCs, after H_2_O_2_ treatment, after H_2_O_2_+miR-206 inhibitor treatment, and H_2_O_2_+miR-206 inhibitor+levamisole treatment, respectively. ^∗^*P* < 0.05, ^∗∗^*P* < 0.01, and ^∗∗∗^*P* < 0.001.

As depicted in Figure 3(a), miR-206 was predicted capable of binding to the 295-302 segments on the 3′-UTR of the Alpl gene. To confirm whether a target relationship existed between miR-206 and Alpl, we designed a dual luciferase reporter vector and performed a dual luciferase reported assay. As shown in Figure 3(b), miR-206 significantly decreased the relative luciferase reporter activity of the wild-type Alpl 3′-UTR, whereas that of the mutant Alpl 3′-UTR did not change, suggesting that miR-206 could directly bind to the 3′-UTR of Alpl.

Furthermore, we detected the Alpl expression level in MSCs after being transfected with miR-206 mimic or miR-206 inhibitor. Both the Q-PCR and western blot results revealed that the Alpl expression was statistically inhibited in the miR-206 mimic group while upregulated in the miR-206 inhibitor group compared to the NC group (Figures 3(c) and 3(d)). Besides, we found that Alpl was lowly expressed in MSCs after H_2_O_2_ treatment and nearly recovered to the base level in H_2_O_2_+miR-206 inhibitor-treated MSCs (Figure 3(e)). Above all, these results strongly indicated that Alpl was a target gene of miR-206.

### 3.4. Inhibition of miR-206 Alleviated the H_2_O_2_-Induced Senescence of Rat MSCs

**Figure 4:**
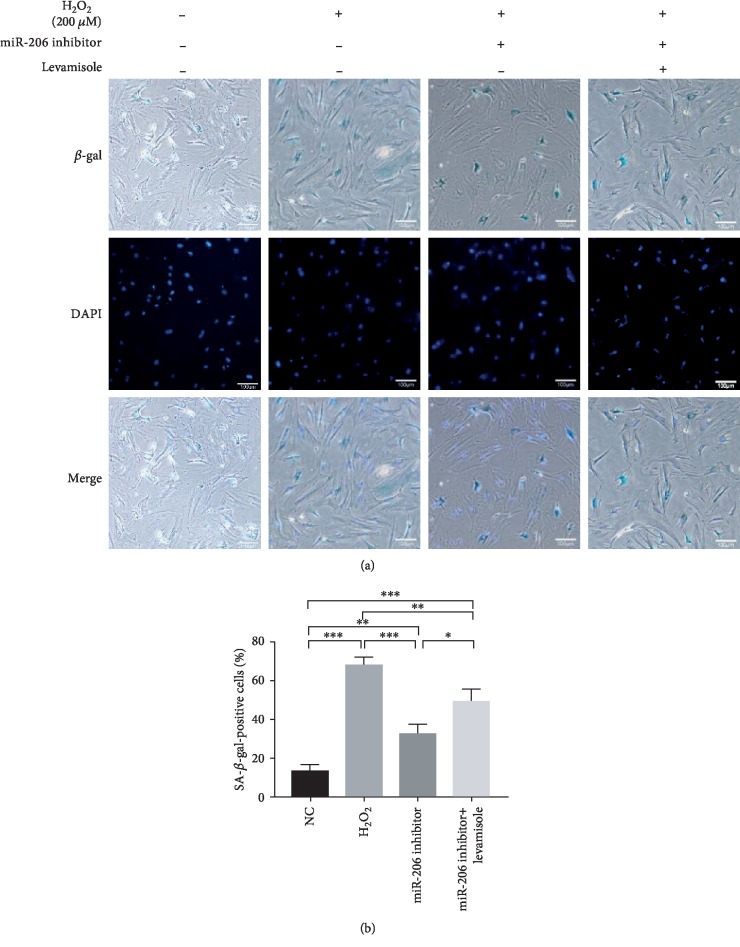
Inhibition of miR-206 alleviated the senescence of rat MSCs. (a) Representative images of SA-*β*-gal staining in the NC group, H_2_O_2_ group, miR-206 inhibitor group, and miR-206 inhibitor+levamisole group. (b) The percentage of SA-*β*-gal staining positive cells in the four groups was statistically analyzed (*n* = 3). ^∗^*P* < 0.05, ^∗∗^*P* < 0.01, and ^∗∗∗^*P* < 0.001.

In view of the above results, we transfected the miR-206 inhibitor in MSCs and performed SA-*β*-gal staining assay. Figures 4(a) and 4(b) showed that the percentage of blue staining positive cells in the miR-206 inhibitor group was significantly reduced compared to that in the H_2_O_2_ group. However, when Alpl expression was suppressed at the same time, the reduced percentage of blue staining positive cells was partially increased. These suggested that inhibition of miR-206 could alleviate the H_2_O_2_-induced MSC senescence via targeting Alpl.

### 3.5. Effects of miR-206 on the Proliferation of Rat MSCs

**Figure 5:**
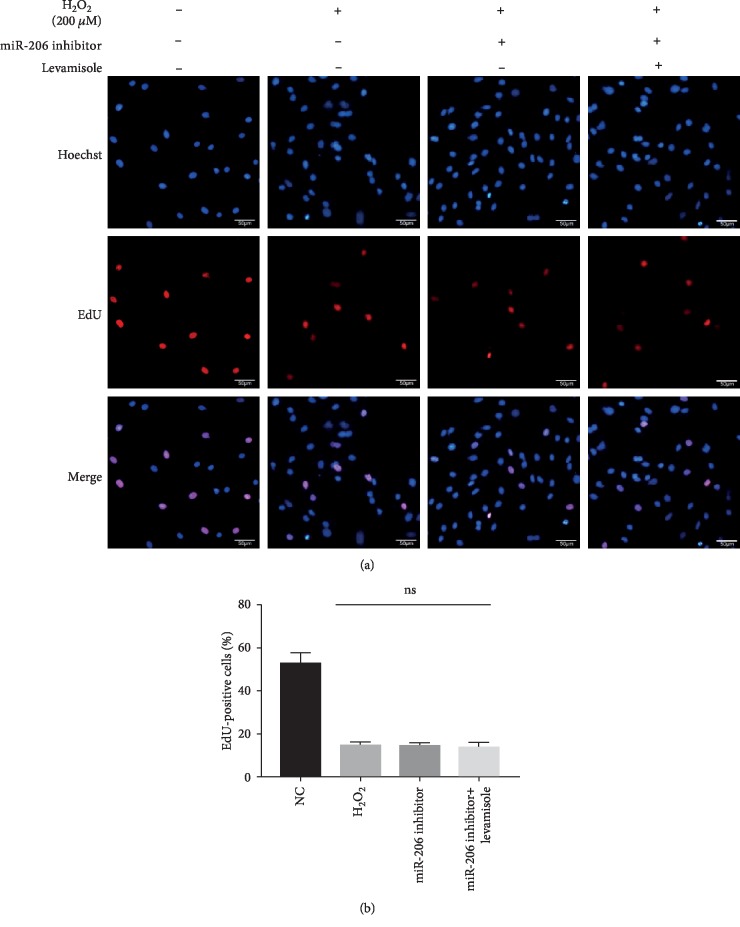
Suppression of miR-206 showed no effect on the proliferation of rat MSCs. (a) Representative images of EdU staining in the NC group, H_2_O_2_ group, miR-206 inhibitor group, and miR-206 inhibitor+levamisole group. (b) The percentage of EdU staining positive cells in the four groups was statistically analyzed (*n* = 3).

Besides, we performed an EdU staining experiment to investigate the effect of miR-206 on the proliferation activity of MSCs. As shown in Figures 5(a) and 5(b), neither addition of miR-206 inhibitor nor supplementation of miR-206 inhibitor and levamisole could repair the severely damaged proliferation activity of MSCs caused by H_2_O_2_, indicating that miR-206 had no effect on the proliferation activity of MSCs.

### 3.6. Repression of miR-206 Protected the Migration Ability and Paracrine Function of Rat MSCs

**Figure 6:**
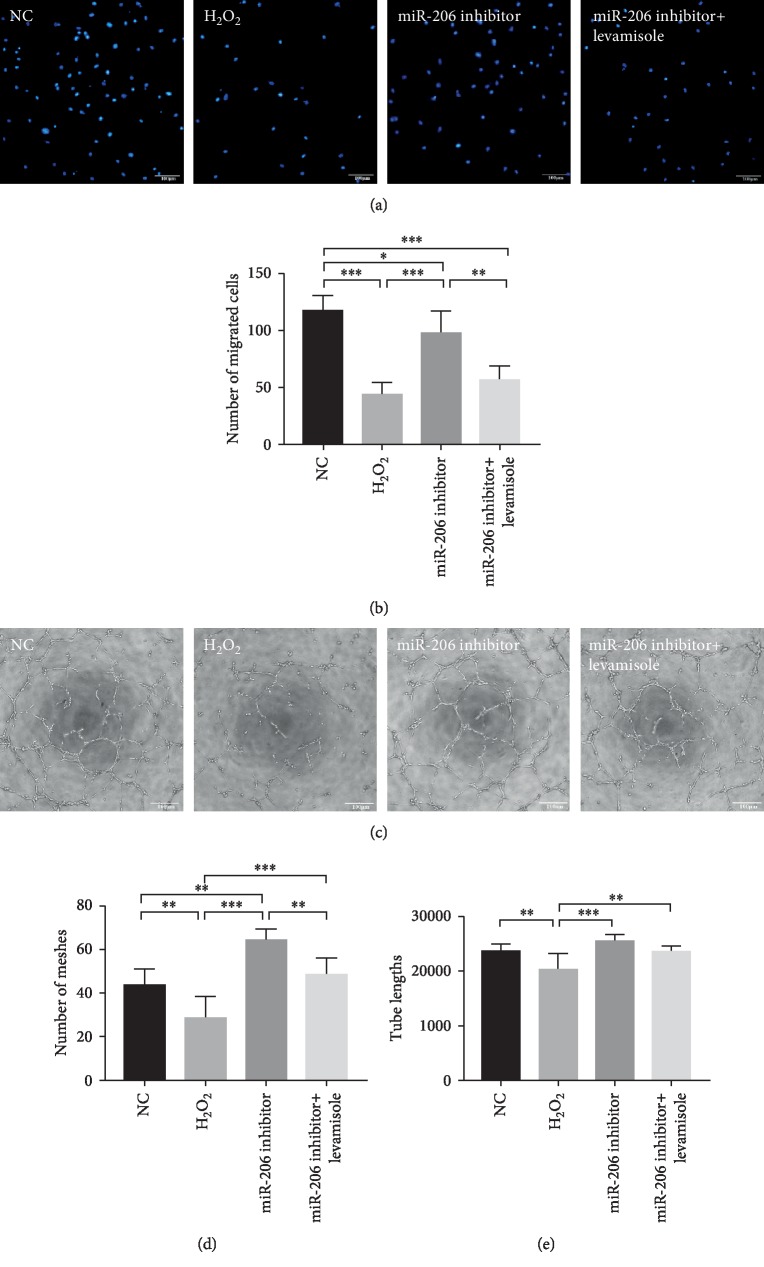
Repression of miR-206 protected the migration and paracrine function of rat MSCs. (a) Representative images of migrated cells in the NC group, H_2_O_2_ group, miR-206 inhibitor group, and miR-206 inhibitor+levamisole group stained with DAPI. (b) Number of migrated cells in the four groups was statistically analyzed (*n* = 4). (c) Representative images of tube formation in the NC group, H_2_O_2_ group, miR-206 inhibitor group, and miR-206 inhibitor+levamisole group. The number of meshes (d) and tube lengths (e) in the four groups was statistically analyzed (*n* = 3). ^∗^*P* < 0.05, ^∗∗^*P* < 0.01, and ^∗∗∗^*P* < 0.001.

In addition, we determined the migration ability of MSCs by transwell assay. Results showed that the migration ability of MSCs after H_2_O_2_ treatment had a significant decrease in contrast to normal MSCs. However, when the expression of miR-206 was inhibited, the impaired migration ability of MSCs was partially alleviated (*P* < 0.001 compared to the H_2_O_2_ group), whereas supplementation of levamisole significantly blocked such positive effects (Figures 6(a) and 6(b)). Further, to explore the paracrine function of rat MSCs, we cocultured HUVEC cells with the supernatants of MSCs in different groups for 24 hours and then performed tube formation assay as described previously [22]. As shown in Figures 6(c)–6(e), compared to the NC group, both the meshes numbers and tube lengths in the H_2_O_2_ group showed a dramatic decrease (*P* < 0.01). However, when the expression of miR-206 was suppressed in the miR-206 inhibitor group, the meshes numbers and tube lengths were obviously increased (*P* < 0.001 compared to the H_2_O_2_ group). Not surprisingly, the enhanced paracrine function was also partially counterbalanced by Alpl inhibitor levamisole.

### 3.7. Downregulation of miR-206 in MSCs Effectively Preserved Cardiac Function in a Rat MI Model

**Figure 7:**
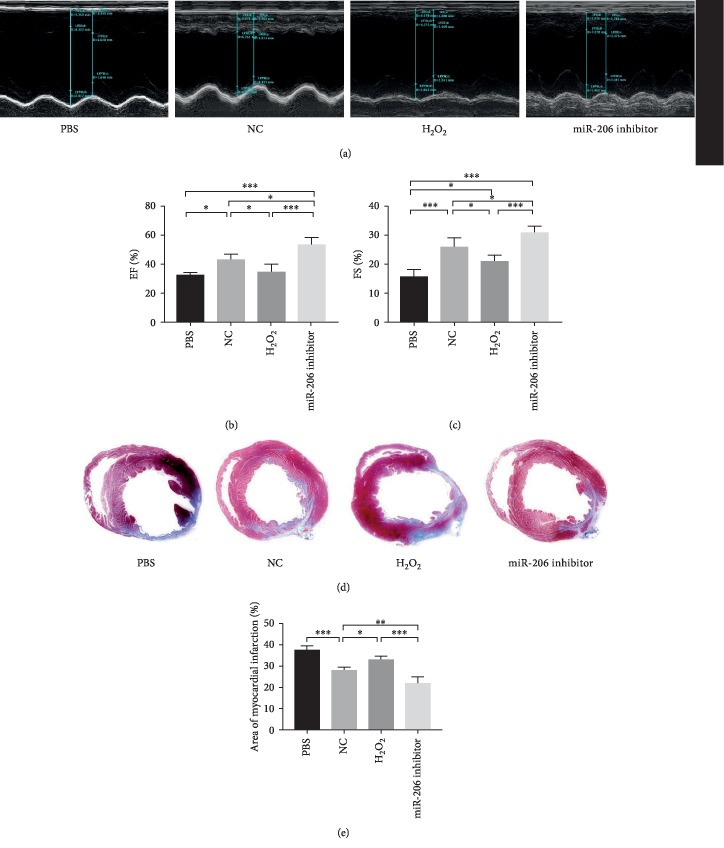
Downregulation of miR-206 in MSCs effectively preserved cardiac function. (a) Representative images of rat heart function detected by echocardiogram in PBS, NC, H_2_O_2_, and miR-206 inhibitor groups at 28 days after MI (*n* = 5). The left ventricular ejection fraction (EF) (b) and fraction shorting (FS) (c) in the four groups were statistically analyzed (*n* = 5). (d) Representative images of Masson trichrome (MT) staining in PBS, NC, H_2_O_2_, and miR-206 inhibitor groups at 28 days after MI. (e) Percentage of fibrotic area calculated and analyzed by ImageJ software (*n* = 5). ^∗^*P* < 0.05, ^∗∗^*P* < 0.01, and ^∗∗∗^*P* < 0.001.

The in vivo therapeutic effects of MSCs in different groups were assessed based on a rat MI model. To evaluate the heart function, the left ventricular ejection fraction (LVEF) and fractional shortening (FS) were measured by echocardiography preoperation and on 28 days postoperation. No significant differences were found among the four groups preoperation (data not shown). However, at 28 days postoperation, the LVEF and FS in the PBS group was significantly decreased, while both the NC group, H_2_O_2_ group, and miR-206 inhibitor group showed rescued heart function. More importantly, the LVEF was even significantly increased in the miR-206 inhibitor group than in the NC group (*P* < 0.05, Figures 7(a)–7(c)).

**Figure 8:**
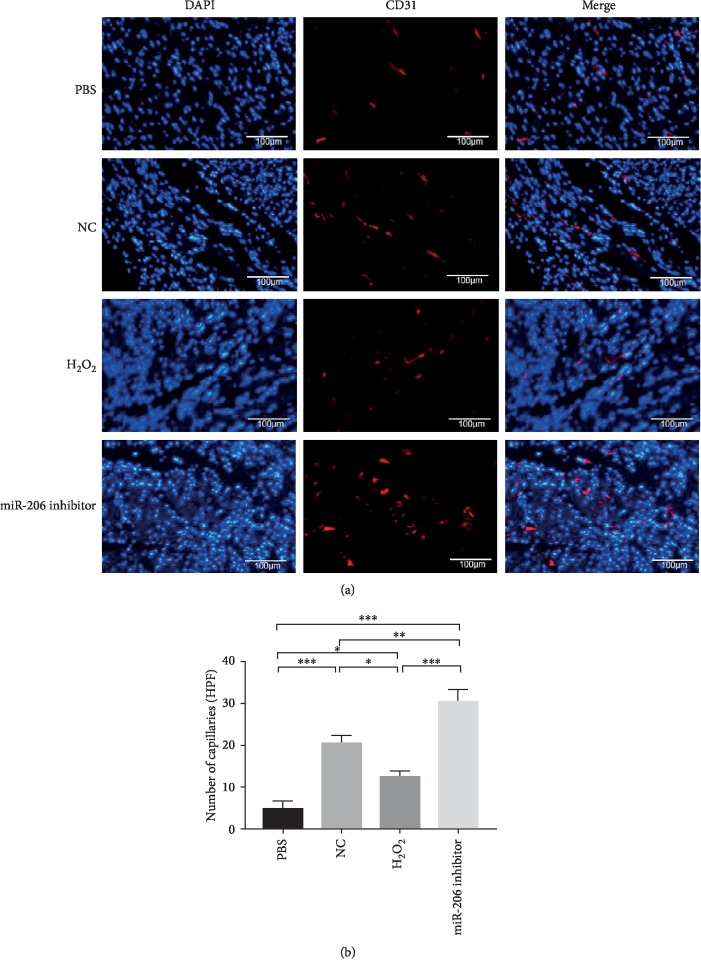
CD31 staining of angiogenesis in rat hearts after MI. (a) Representative images of neovascular in rat heart infarction area in PBS, NC, H_2_O_2_, and miR-206 inhibitor groups detected by CD31 staining (*n* = 3). (b) CD31-positive vessels in the four groups were statistically analyzed (*n* = 3). ^∗^*P* < 0.05, ^∗∗^*P* < 0.01, and ^∗∗∗^*P* < 0.001.

After 28 days, the rat hearts were collected and sliced for further immunohistochemical staining. As depicted in Figures 7(d) and 7(e), MT staining showed that the fibrosis area in the miR-206 inhibitor group was significantly reduced compared to both PBS, NC, and H_2_O_2_ groups. Besides, CD31 staining showed that angiogenesis in the cardiac infarct area was also significantly increased in the miR-206 inhibitor group compared to the other three groups (Figures 8(a) and 8(b)). These data demonstrated that inhibition of miR-206 in senescent MSCs could effectively protect their potential for myocardial infarction therapy in a rat MI model.

## 4. Discussion

In the present study, we demonstrated that miR-206 was upregulated in the senescent MSCs induced by H_2_O_2_ treatment. And by modulating its putative target gene Alpl, miR-206 played the role of regulator in the senescent process of MSCs. Besides, we examined that inhibition of miR-206 could partially improve the migration ability and paracrine function of senescent MSCs, thereby protecting their therapeutic potential in the treatment of rat MI.

Transplantation of MSCs as an autologous stem cell for cell-based therapy and tissue engineering is promising in regenerative medicine. However, replicative senescence and pathological conditions, such as serum deprivation, hypoxia, and oxidative stress inevitably compromised the therapeutic potential of MSCs by inducing the cells into senescence or apoptosis. For example, researchers have revealed that, caused by the occurrence of replicative senescence, MSCs in early passages showed enhancer active proliferation and shorter doubling time compared to MSCs in late passages [23–25]. Besides, Palumbo et al. have confirmed that oxidative stress-induced senescence could reduce the regenerative potential of MSCs and lead to increased sensitivity of senescent MSCs to apoptotic cell death [26]. Consequently, Rauscher et al. have found that transplantation of MSCs derived from older donors appeared to be less effective compared to younger counterparts [27].

Reports have shown that H_2_O_2_ treatment of cultured cells is a commonly used model to test oxidative stress, thus playing an important role in the induction of stress-induced premature senescence [28, 29]. In accordance with previous reports, we successfully induced senescent MSCs by H_2_O_2_ treatment at a concentration of 200 *μ*M, which was evidenced by the SA-*β*-gal staining assay, EdU staining assay, apoptosis detection assay, and ELISA assay.

Previous studies on miR-206 were mainly focused on its association with the pathogenesis of human cancers. And it was confirmed that upregulation of miR-206 could inhibit the proliferation and migration, block the cell cycle, and promote apoptosis in several cancers cells, including gastric cancer [30], breast cancer [31], and cervical cancer [11]. In our previous study, we firstly reported the association between miR-206 and MSCs. Specifically, miR-206 was upregulated in MSCs under hypoxic condition, and inhibition of miR-206 had antiapoptotic and migration-promoting effects on MSCs [32]. Later, Chen et al. reported that miR-206 could inhibit the osteogenic differentiation of MSCs by targeting glutaminase [33]. Recently, miR-206 was reported downregulated in response to oxidative stress in cardiomyocytes and I/R in the heart, and exogenous miR-206 expression could reduce injury whereas endogenous miR-206 could promote survival of cardiomyocytes during I/R [10]. Documents also revealed that miR-206 was pathological upregulated in a rat heart model of MI and regulated the apoptotic cell death of cardiomyocytes [34]. Besides, researchers also observed that after acute myocardial infarction (AMI), the activity of miR-1 and miR-206 can be increased by histamine, leading to a decrease in autophagy activation and cell apoptosis under hypoxia and AMI conditions by acting on Atg13 [35].

In this study, we detected that miR-206 was significantly upregulated in H_2_O_2_-induced senescent MSCs. Since microRNAs play a regulatory role by combining to the 3′UTR of the target gene, therefore, we aimed to determine the downstream mRNAs modulated by miR-206. Through the database (http://TargetScan.com), we found that miR-206 was capable of binding on the 3′-UTR region of Alpl. It has been reported that Alpl could prevent bone ageing sensitivity by specifically regulating the senescence and differentiation of MSCs [36]. In view of this, we then performed luciferase reporter assay, Q-PCR, and western blot analysis and validated the targeting relationship of miR-206 and Alpl.

Senescent MSCs have shown reduced multipotency, impaired proliferation activity, and weakened paracrine ability; thus, the regenerative potential of MSCs for the desired therapeutic effects was greatly limited [37, 38]. Consistent with this, we observed decreased proliferation, migration, and paracrine ability in H_2_O_2_-induced senescent MSCs. Subsequently, we demonstrated that inhibition of miR-206 played an important role in mediating the above cascade changes in senescent MSCs by targeting Alpl. Moreover, the HUVEC angiogenesis in the miR-206 inhibitor group was even superior to that in the NC group. Similarly, an in vitro study observed that CCL19 high-expressed SW1116 supernatant was able to inhibit the angiogenesis of HUVEC through promoting miR-206, whereas CCL19 low-expressed SW620 supernatant can promote HUVEC angiogenesis [22]. Besides, Wang et al. have reported that amphiregulin enhanced VEGF-A production in human chondrosarcoma cells and promoted angiogenesis by inhibiting miR-206 via the FAK/c-Src/PKCd pathway [39]. Moreover, Cai et al. have shown that miR-206 could diminish the expression and production of VEGF-A, subsequently reducing tumor angiogenesis in renal carcinoma [40].

Transplantation of MSCs has been widely used in the treatment of certain tissue injuries such as ischemic heart failure and hind-limb ischemia [41]. However, senescence would reduce the regenerative potential of MSCs and is one of the main reasons for increased susceptibility of senescent MSCs to apoptotic cell death under ischemic conditions [26]. In this study, we found that the effect of senescent MSCs on myocardial infarction treatment was worse than that of normal MSCs, but inhibiting the expression of miR-206 in senescent MSCs showed improved therapeutic effect. Besides, we conducted MT staining and CD31 immunofluorescent staining, a sort of endothelial cell-specific surface marker used to determine the neovascular. And we observed a reduced myocardial infarction area and increased angiogenesis in the miR-206 inhibitor group than in other groups.

## 5. Conclusion

The underlying molecular mechanisms required to illustrate the oxidative stress-induced premature senescence of MSCs is far from being understood. Here, we demonstrated that downregulation of miR-206 in MSCs can alleviate H_2_O_2_-induced senescence and partially resist the migration ability and paracrine function impairment, as well as protect the therapeutic efficacy in MI treatment. Therefore, application of the protective effects of miR-206 in senescent MSCs may provide new strategies in autologous stem cell transplantation therapy.

## Figures and Tables

**Table 1:** Primer sequences used for Q-PCR.

Gene	Forward primer (5′-3′)	Reverse primer (5′-3′)
GAPDH	ACAGCAACAGGGTGGTGGAC	TTTGAGGGTGCAGCGAACTT
Alpl	GGAGATGGATGAGGCCATCG	CCATGATGGTTGCAGGGTCT
Tel	GGTTTTTGAGGGTGAGGG- TGAGGGTGAGGGTGAGGG	TCCCGACTATCCCTATCCCTA- TCC CTATCCCTATCCCTA
AT1	ACGTGTTCTCAGCATCGA -CCGCTACC	AGAATGATAAGGAAAGGG- AACAAGAAGCCC

## Data Availability

The data used to support the findings of this study are available from the corresponding authors upon request.
